# Dietary and lifestyle oxidative balance scores are independently and jointly associated with nonalcoholic fatty liver disease: a 20 years nationally representative cross-sectional study

**DOI:** 10.3389/fnut.2023.1276940

**Published:** 2023-10-18

**Authors:** Yuanbin Liu, Mingkai Chen

**Affiliations:** Department of Gastroenterology, Renmin Hospital of Wuhan University, Wuhan, China

**Keywords:** nonalcoholic fatty liver disease, NHANES, advanced liver fibrosis, oxidative stress, antioxidant

## Abstract

**Background:**

Oxidative stress is an important contributor to the progression of nonalcoholic fatty liver disease (NAFLD), but whether dietary and lifestyle pro- and antioxidants may have combined or independent effects on NAFLD, and advanced liver fibrosis (AHF) remains unclear. We aimed to elucidate the relationship between a well-established oxidative balance score (OBS) and NAFLD/AHF.

**Methods:**

This was a cross-sectional study. We included adult participants with complete data from the National Health and Nutrition Examination Survey 1999–2018. Survey-weighted adjusted multivariate regression analyses were used to examine the association of all OBS with NAFLD/AHF. A combination of restricted cubic splines, mediation analysis, stratified analysis, and sensitivity analysis were used to further elucidate these associations.

**Results:**

We included 6,341 eligible adult participants with prevalence of NAFLD and AHF of 30.2 and 13.9%, respectively. In the fully adjusted model, the highest quartile of OBS, dietary OBS, and lifestyle OBS were associated with 65, 55, and 77% reduced risk of NAFLD, respectively, compared with the reference population, respectively. However, all OBS were not associated with the risk of AHF. All OBS were nonlinearly associated with risk of NAFLD and had a more pronounced reduced risk for OBS, dietary OBS, and lifestyle OBS after exceeding 26, 21, and 5 points, respectively. OBS may exert a protective effect indirectly through inflammation, oxidative stress, and glycolipid metabolism markers. Stratification and sensitivity analyses demonstrate the robustness of our findings.

**Conclusion:**

All OBS were nonlinearly and negatively associated with NAFLD risk. These effects may exert indirectly through inflammation, oxidative stress, and glycolipid metabolism markers.

## Introduction

Nonalcoholic fatty liver disease (NAFLD) is currently the most common chronic liver disease globally, with a course ranging from simple hepatic steatosis to the more severe form, nonalcoholic steatohepatitis (NASH), and a small percentage can progress to cirrhosis, liver cancer, and lead to death (1). The diagnosis of NAFLD is determined by the evidence of the presence of hepatic steatosis (accumulation of more than 5% fat in hepatocytes) confirmed by noninvasive markers/imaging/biopsy and the exclusion of secondary chronic liver injury (e.g., excessive alcohol consumption, viral hepatitis, etc.) (2). Generally, one in three adults worldwide may suffer from NAFLD, and its incidence continues to increase at an alarming rate (3). Better understanding of NAFLD/NASH and risk stratification by recognizing modifiable risk factors will help alleviate this public health burden.

Although the pathogenesis of NAFLD is still not fully understood, the accepted perspective is the ‘multiple hit’ hypothesis. Multiple factors including environmental exposures, unhealthy diet/lifestyle, and genetics/epigenetics have been shown to contribute to the following metabolic dysregulation, immune dysregulation, inflammation, and fibrosis through a variety of pathophysiological mechanisms (4). Oxidative stress caused by events such as mitochondrial dysfunction and endoplasmic reticulum stress has been well defined as an important hallmark for the progression from simple steatosis to NASH with more severe inflammation and fibrosis (5). Therefore, modulation of reactive oxygen species (ROS) production is currently an important research direction for therapies targeting NAFLD, especially NASH. Currently, several agents targeting anti-oxidative stress including vitamin E have shown improvement in NASH in preclinical experiments and clinical trials (6).

However, the impact of comprehensive dietary/lifestyle modifiable interventions related to oxidative stress on NAFLD and more severe cohorts such as those with advanced fibrosis is still lacking. Awareness of the impact of these modifiable risk factors and timely intervention may be of great value in the prevention of NAFLD progression and remission of NASH. To fill this knowledge gap, we employ a previously well-established oxidative balance score (OBS) in a nationally representative survey, the National Health and Nutrition Examination Survey (NHANES), to examine its effects on NAFLD and advanced liver fibrosis (AHF) for the first time.

## Method

### Study design and population

The NHANES is a series of nationally representative, multi-stage, cross-sectional surveys with complex probability sampling. It was reviewed and approved by the NCHS Ethics Review Board, and informed consent was obtained from all participants. Further information can be found on the official website.

**Figure 1:**
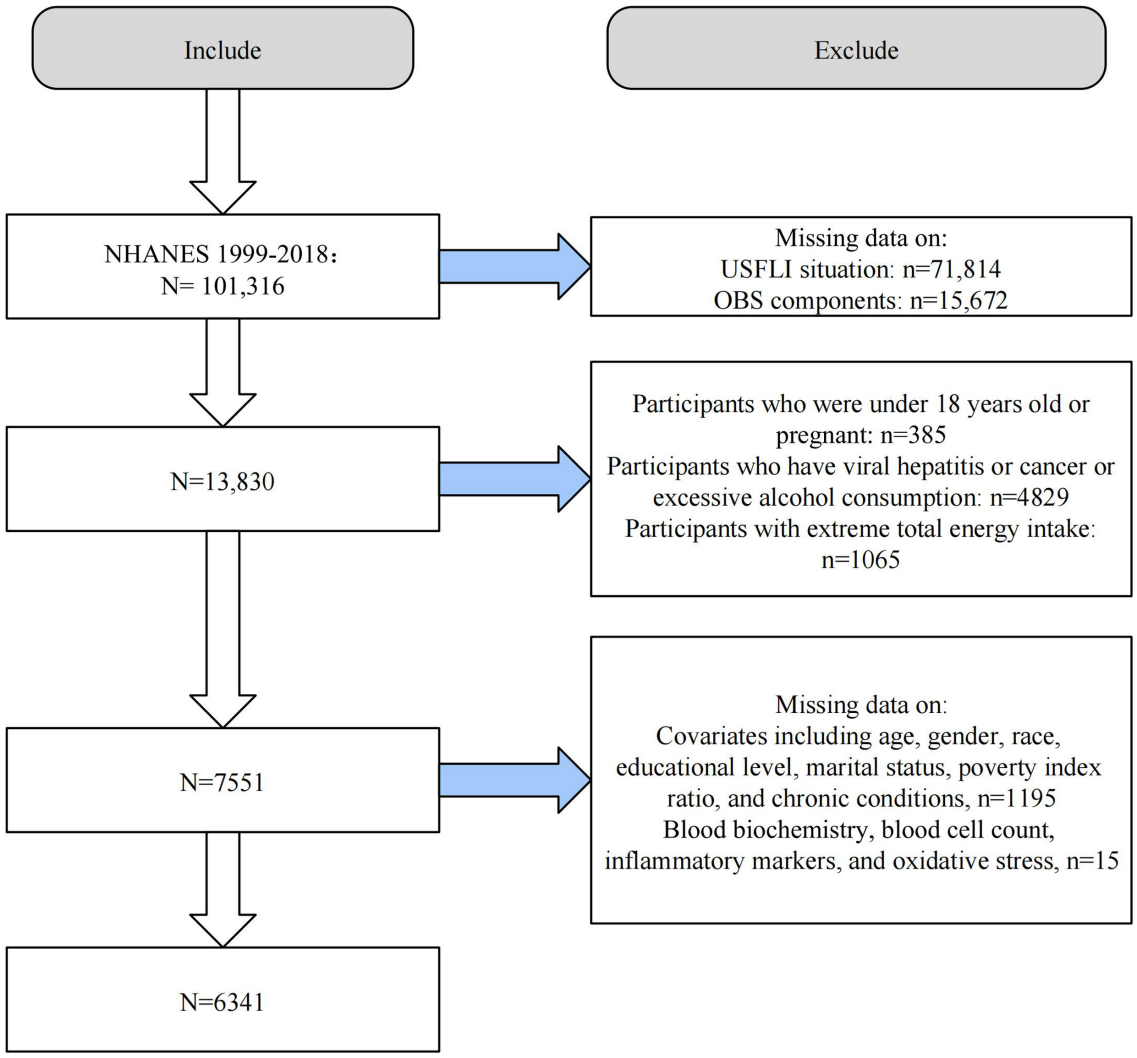
Flow chart of study population selection. USFLI, US fatty liver index; OBS, oxidative balance score; NHANES, the National Health and Nutrition Examination Survey.

We conducted the analysis using ten consecutive cycles of NHANES 1999–2018. From the initial 101,316 individuals, we first excluded all those missing data for the US Fatty Liver Index (USFLI) (*n* = 71,814) and OBS components (*n* = 15,672). Second, we excluded those younger than 18 years or pregnant (*n* = 385), those with other liver diseases such as chronic hepatitis, excessive alcohol intake (≥3 drinks/day for men and ≥2 drinks/day for women), and cancer (*n* = 4,829), and those with extreme total energy intake (*n* = 1,065). Finally, we excluded populations with missing data on included covariates (*n* = 1,195) and those with missing mediating variables (*n* = 15). The final number of eligible adult participants was 6,341 (Figure 1).

### Composition of OBS

We calculated the OBS based on previous well-established studies (7–9). The OBS used in this study consisted of 16 dietary factors and 4 lifestyle measures that have previously been shown to be associated with oxidative stress (Supplementary Table S1). These components were divided into pro-oxidants and antioxidants based on their effect on oxidative stress. Specifically, pro-oxidants consisted of dietary total fat, dietary iron intake, alcohol consumption, body mass index (BMI), and serum cotinine, while antioxidants included dietary fiber, carotenoids, riboflavin (vitamin B2), niacin, vitamin B6, total folate, vitamin B12, vitamin C, vitamin E, calcium, magnesium, zinc, copper, selenium intake, and physical activity. Dietary intake information was obtained from two 24 h dietary review questionnaires consisting of a 24 h dietary recall interview conducted at a Mobile Examination Center and a telephone follow-up 3–10 days later through the USDA’s Automated Multiple-Pass Method. The average of the two 24 h recall dietary interviews was used to proxy its intake. The OBS components and intakes for food or beverage consumed in the 24 h prior to the interview were collected and recorded by the NHANES computer-assisted dietary interview system. Instead, dietary nutrient intake was assessed according to the University of Texas Food Intake Analysis System and the USDA’s Food and Nutrient Database for Dietary Studies. We did not include intake of nutrients from dietary supplements and medications based on the previous study as these data were lacking or poorly provided in full in many cycles (9). Notably, we used serum nicotine as a proxy for smoking based on previous studies, as it incorporated both active and passive smoking levels. More information on the data collection can be found in the previous study (9).

Our allocation scheme is described in Table 1. Most of the components were assigned according to their sex-specific tertiles, with antioxidants assigned 0, 1, and 2 points from tertile 1 to 3, respectively, and pro-oxidants assigned the reverse. Instead, alcohol consumption was assigned a score based on recognized intake criteria (0 point for men ≥2 drinks/d and ≥1 drinks/d for women, 1 point for men <2 drinks/d and <1 drinks/d for women; and 2 points for <12 drinks/year). In addition, we divided OBS into dietary and lifestyle OBS according to the source of the components to explore their combined and independent effects on NAFLD/AHF, respectively.

**Table 1:** OBS allocation scheme.

	Male			Female		
	0	1	2	0	1	2
Dietary OBS components						
Dietary fiber (g/d)	<12.6	<19.7	≥19.7	<10.1	<16.35	≥16.35
Carotene (RE/d)	<99.04	<306.64	≥306.64	<98.25	<383.96	≥383.96
Vitamin B2(mg/d)	<1.79	<2.69	≥2.69	<1.34	<2.02	≥2.02
Niacin (mg/d)	<20.68	<29.75	≥29.75	<14.528	<21.86	≥21.86
Vitamin B6 (mg/d)	<1.59	<2.40	≥2.40	<1.13	<1.77	≥1.77
Total folate (mcg/d)	<316	<492	≥492	<251	<389	≥389
Vitamin B12 (mcg/d)	<3.36	<6.2	≥19.13	<2.22	<4.22	≥4.22
Vitamin C (mg/d)	<42.5	<113.21	≥113.21	<38.01	<98.5	≥98.5
Vitamin E (ATE) (mg/d)	<5.82	<9.43	≥9.43	<4.54	<7.52	≥7.52
Calcium (mg/d)	<6.46	<1072.5	≥1072.5	<499.5	<849	≥849
Magnesium (mg/d)	<257	<361.28	≥361.28	<187	<283.43	≥283.43
Zinc (mg/d)	<9.75	<15.1	≥15.1	<6.73	<10.73	≥10.73
Copper (mg/d)	<1.12	<1.57	≥1.57	<0.85	<1.28	≥1.28
Selenium (mcg/d)	<94.94	<141.85	≥141.85	<67.83	<99.5	≥99.5
Total fat (g/d)	≥107.42	<107.42	<69.83	≥75.775	<75.775	<50.965
Iron (mg/d)	≥19.165	<19.165	<12.88	≥14.315	<14.315	<9.65
Lifestyle OBS components						
Physical activity (MET-minute/week)	<417.9	<1136.8	≥1136.8	<274	<846	≥846
Alcohol (drinks/d)	≥2 drinks/d	<2 drinks/d	<12 drinks/year	≥1 drinks/d	<1 drinks/d	<12 drinks/year
Body mass index (kg/m2)	≥29.16	<29.16	<25.55	≥28.63	<28.63	<23.74
Cotinine (ng/mL)	≥1.08	<1.08	<0.038	≥0.171	<0.171	<0.035

OBS, oxidative balance score.

### Definition of NAFLD/AHF

Suspected NAFLD was defined according to the USFLI, which was developed using the NHANES database, which has moderately improved accuracy compared to the FLI in the multi-ethnic US population (10). USFLI was developed based on ethnicity, age, gamma glutamyltransferase (GGT), waist circumference (WC), fasting insulin, and fasting glucose with the following formula: USFLI = (e^−0.8073 × non-Hispanic black + 0.3458 × Mexican American + 0.0093 × age + 0.6151 × ln (GGT) + 0.0249 × WC + 1.1792 × ln (insulin) + 0.8242 × ln (glucose)−14.7812^)/(1 + e^−0.8073 × non-Hispanic black + 0.3458 × Mexican American + 0.0093 × age + 0.6151 × ln (GGT) + 0.0249 × WC + 1.1792 × ln (insulin) + 0.8242 × ln (glucose)−14.7812^) × 100. A USFLI ≥30 was considered to have putative NAFLD with an area under the receiver operating characteristic curve of 0.80 (10). AHF was defined as NAFLD fibrosis score (NFS) > 0.676 or fibrosis 4 index (FIB-4) > 2.67 or aspartate aminotransferase (AST)/platelet ratio index (APRI) > 1 in the presence of NAFLD (11). NFS has been validated to diagnose AHF non-invasively and accurately using the formula: NFS = −1.675 + 0.037 × age + 0.094 × BMI + 1.13 × impaired fasting glycemia or diabetes (yes = 1, no = 0) + 0.99 × AST/alanine aminotransferase (ALT) − 0.013 × platelet −0.66 × albumin (12). The formula for FIB-4 is: FIB-4 = (age × AST)/(Platelet counts × (SQRT(ALT))) (13). The formula for APRI is: APRI = ([AST/upper limit of normal (ULN)]/Platelet counts) × 100 (14). In the 1999–2000 cycle of NHANES, AST = 40 U/L was used as ULN, and 33 U/L was used as ULN in subsequent years (15).

### Covariates

Based on previous relevant studies, we selected several possible covariates, including age, gender (male or female), ethnicity (Mexican American, non-Hispanic black, non-Hispanic white, other Hispanic, or other races), education (<high school, high school, or >high school), family income to poverty ratio (PIR), marital status, total energy intake, and chronic comorbid conditions including a range of other cardiometabolic diseases, i.e., diabetes, hypertension, and self-report cardiovascular disease (CVD) (including coronary heart disease, stroke, heart attack, congestive heart failure, and angina). The criteria for diagnosing hypertension was that a doctor says someone has high blood pressure, is taking antihypertensive medication, or that their blood pressure values are in the hypertensive range (16). Diabetes was diagnosed based on if a doctor said that a person has diabetes, abnormal blood glucose/glucose tolerance test, or was taking related medication (17).

### Mediation variables

**Figure 2:**
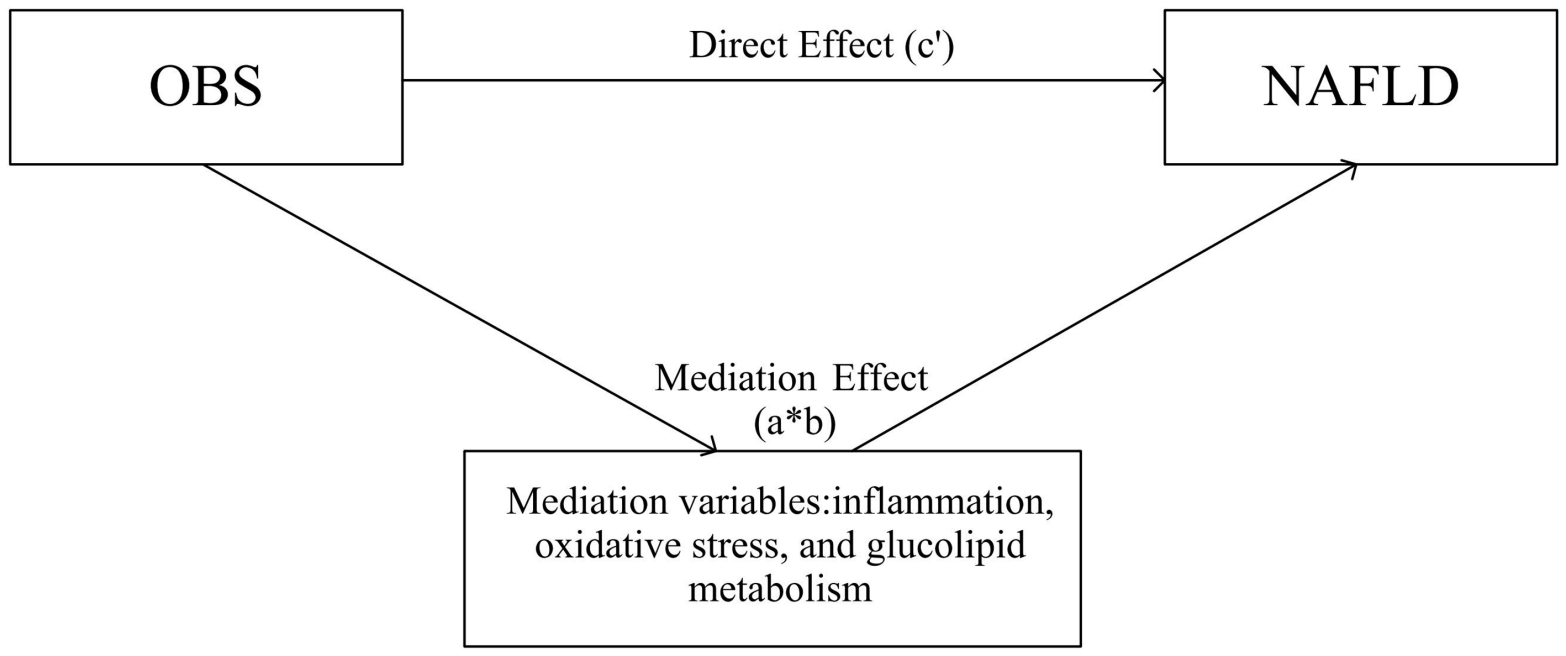
Schematic diagram of the mediation effect. OBS, oxidative balance score; NAFLD, nonalcoholic fatty liver disease.

We speculated that OBS may have indirect effects on NAFLD through several other pathways. First, oxidative stress-related markers may be important mediators, and second, given the close association of oxidative stress with inflammation and metabolism, we considered the potential mediating effects of these markers (Figure 2). To investigate whether OBS acts indirectly on NAFLD through mediators related to inflammation, oxidative stress, and glycolipid metabolism, we performed a mediation analysis. We selected several important biomarkers of these relevant aspects in NAFLD based on the literature, including blood neutrophils (18), ALT/AST (19), serum albumin (20), uric acid (UA) (21), bilirubin (22), GGT (23), triglycerides (TG) (24), total cholesterol (TC) (24), high-density lipoprotein (HDL)-cholesterol (24), and fasting blood glucose (25). Detailed laboratory tests and data collection instruments are publicly available.^1^

### Statistical analysis

All analyses were conducted using EmpowerStats software and R version 4.1.3. Due to the complex design of NHANES, we appropriately weighted our data analysis according to the NHANES survey reporting guidelines for the survey. For continuous variables, we used the survey-weighted mean (95% confidence interval [CI]), and the value of *p* was calculated by survey-weighted linear regression. For categorical variables, we used survey-weighted percentages (95% CI), and value of *p*s were calculated by survey-weighted Chi-square tests. We used multivariate logistic regression analysis to examine the association between OBS and NAFLD/AHF. We constructed three multivariate regression models: first, we constructed model 1 (unadjusted model), which did not adjust for any covariates; model 2 adjusted for age, sex, race, marital status, education level, and energy intake (kcal/day); model 3 was based on model 2 and added adjustments for diabetes, hypertension, and CVD to model 2. Restricted cubic splines (RCS) were used to describe the nonlinear relationship between the OBS and NAFLD/AHF. The curve fitting term is defined by the RCS function from the rms package, and the degrees of freedom (or knots) are determined according to the magnitude of the P for nonlinear value.

To investigate whether OBS may influence the risk of NAFLD through other pathways, we performed a mediation analysis by selecting possible markers as mediating variables. The total effect of OBS on the risk of NAFLD (*c*) includes the direct effect of OBS (*c*′) and the indirect effect through mediating variables (a*b), and *c* = *c*′ + a*b. For the markers of inflammation, oxidative stress, and glycolipid metabolism included in this study, we performed individual mediator analyses based on previous studies and calculated direct effects in the association of OBS with risk of NAFLD and indirect effects through these markers. We calculated the proportion of the total effect mediated by the included mediating variables. All mediation analyses were adjusted for all covariates. In addition, stratified and sensitivity analyses were performed to test the consistency of the findings across subgroups and the stability of the results.

## Results

### Baseline characteristics

6,341 adult participants (representing a weighted US population of 129,919,267) were included in our study. 1,917 and 266 individuals were diagnosed with NAFLD and AHF (prevalence of 30.2 and 13.9%, respectively). We divided the quartiles according to OBS, and all indicators including PIR, gender, race, education level, marital status, daily energy intake, presence of diabetes, and hypertension were significantly different (all *p* < 0.05), except for age and presence of CVD, which did not differ between quartiles. As the quartiles of OBS gradually increased, participants were wealthier, had a higher proportion of women, a higher proportion of non-Hispanic whites, a higher level of education, a higher proportion of singles, a higher daily energy intake, and a lower prevalence of diabetes and hypertension (Table 2). We also divided the population according to the presence of NAFLD and AHF. Those with NAFLD differed significantly on all covariates except for PIR and daily energy intake, which did not differ from those without NAFLD. OBS (both dietary and lifestyle OBS) were significantly lower in the NAFLD population (all *p* < 0.0001). However, while OBS and dietary OBS differed in patients with AHF compared to non-AHF (1,917 patients with NAFLD had accessible diagnostic data), baseline lifestyle OBS showed no difference in the population (*p* = 0.786) (Supplementary Tables S2, S3).

**Table 2:** Baseline data according to OBS quartiles.

	Q1	Q2	Q3	Q4	Value of *p*
Mean ± SD	15.408 ± 2.908	22.671 ± 1.677	27.987 ± 1.395	34.674 ± 2.896	
Sample *N*	*n* = 1,475	*n* = 1,602	*n* = 1,438	*n* = 1826	
Weighted *N*	*N* = 32,585,939	*N* = 31,950,344	*N* = 28,849,243	*N* = 36,533,742	
OBS Dietary	11.662 (11.425,11.899)	18.445 (18.281,18.609)	23.298 (23.139,23.457)	28.980 (28.785,29.176)	<0.0001
OBS Lifestyle	3.541 (3.435,3.647)	4.166 (4.049,4.282)	4.649 (4.529,4.769)	5.467 (5.384,5.551)	<0.0001
Age	46.876 (45.533,48.220)	48.310 (47.136,49.484)	48.218 (46.291,50.146)	46.838 (45.649,48.027)	0.1248
PIR	2.929 (2.805,3.053)	3.307 (3.176,3.438)	3.458 (3.302,3.614)	3.506 (3.364,3.648)	<0.0001
Gender					0.0050
Male	55.535 (51.278,59.713)	55.786 (52.545,58.979)	51.776 (47.847,55.683)	47.550 (44.240,50.883)	
Female	44.465 (40.287,48.722)	44.214 (41.021,47.455)	48.224 (44.317,52.153)	52.450 (49.117,55.760)	
Race					<0.0001
Mexican American	5.490 (4.437,6.774)	5.413 (4.268,6.843)	5.316 (4.176,6.746)	5.336 (4.207,6.746)	
Non-Hispanic Black	14.535 (11.710,17.904)	10.090 (8.308,12.203)	7.057 (5.555,8.927)	4.991 (3.979,6.244)	
Non-Hispanic White	67.538 (63.187,71.606)	74.469 (70.455,78.107)	77.732 (74.237,80.874)	79.492 (76.795,81.949)	
Other Hispanic	5.484 (3.556,8.369)	4.842 (2.684,8.583)	3.158 (2.144,4.629)	3.476 (2.496,4.823)	
Other race	6.952 (4.537,10.512)	5.187 (3.919,6.836)	6.737 (5.010,9.001)	6.705 (5.212,8.587)	
Education level					<0.0001
Under high school	6.091 (4.799,7.703)	5.564 (3.682,8.323)	2.943 (2.036,4.237)	2.572 (1.954,3.379)	
High school	40.855 (36.428,45.436)	36.273 (32.559,40.158)	28.498 (24.296,33.108)	22.072 (19.058,25.412)	
More than high school	53.054 (48.394,57.662)	58.164 (54.258,61.969)	68.560 (64.019,72.771)	75.356 (71.808,78.591)	
Marital					0.0010
Single	67.915 (64.192,71.424)	76.400 (73.108,79.404)	73.157 (69.382,76.624)	75.228 (71.497,78.618)	
No single	32.085 (28.576,35.808)	23.600 (20.596,26.892)	26.843 (23.376,30.618)	24.772 (21.382,28.503)	
Daily energy intake(kcal/day)	1617.133 (1552.994,1681.271)	1927.349 (1877.948,1976.750)	2089.965 (2025.818,2154.112)	2307.397 (2249.890,2364.905)	<0.0001
Diabetes					0.0008
No	86.982 (84.206,89.333)	86.471 (83.601,88.905)	89.566 (87.007,91.669)	92.149 (90.125,93.786)	
Yes	13.018 (10.667,15.794)	13.529 (11.095,16.399)	10.434 (8.331,12.993)	7.851 (6.214,9.875)	
Hypertension					<0.0001
No	60.749 (56.496,64.845)	60.319 (56.379,64.129)	64.278 (60.048,68.297)	72.997 (68.969,76.679)	
Yes	39.251 (35.155,43.504)	39.681 (35.871,43.621)	35.722 (31.703,39.952)	27.003 (23.321,31.031)	
CVD					0.1491
No	91.714 (89.477,93.511)	91.323 (89.526,92.837)	93.198 (90.873,94.964)	93.962 (91.764,95.601)	
Yes	8.286 (6.489,10.523)	8.677 (7.163,10.474)	6.802 (5.036,9.127)	6.038 (4.399,8.236)	

OBS, oxidative balance score; SD, standard deviation; PIR, family income to poverty ratio; CVD, cardiovascular disease. For continuous variables: survey-weighted mean (95% CI), value of *p* was by survey-weighted linear regression. For categorical variables: survey-weighted percentage (95% CI), value of *p* was by survey-weighted Chi-square test.

### Multivariate regression analysis

Three models were constructed to explore the relationship between OBS and NAFLD/AHF when used as continuous or categorical variables. Model 1 was an unadjusted model, model 2 was adjusted for age, sex, race, marital status, education level, and daily energy intake, and model 3 was adjusted for all covariates. In all three models, OBS (continuous) (including dietary OBS and lifestyle OBS) was negatively associated with the risk of NAFLD (all *p* < 0.0001). When OBS was used as a categorical variable, the risk of NAFLD gradually decreased with increasing quartiles in all models (P for trend <0.0001). In the fully adjusted model, OBS was associated with a 65% lower risk of NAFLD in Q4 compared to Q1 (reference) (odds ratio [OR] (95% CI) = 0.352 (0.260, 0477), *p* < 0.0001). Similarly, in model 3, dietary OBS and lifestyle OBS in Q4 compared with Q1 reduced the risk of NAFLD by 55 and 77%, respectively, (all *p* < 0.0001) (Table 3). However, OBS (continuous and categorical) was only associated with the risk of AHF in the unadjusted model. There was no association with the risk of AHF in the adjusted models (all *p* > 0.05) (Supplementary Table S4).

**Table 3:** Multivariate regression analysis of OBS and the risk of NAFLD.

	Model 1 OR (95% CI), *P*	Model 2 OR (95% CI), *P*	Model 3 OR (95% CI), *P*
OBS	0.960 (0.950, 0.971) <0.0001	0.945 (0.933, 0.957) <0.0001	0.947 (0.934, 0.960) <0.0001
*OBS Quartile*			
Q1	ref.	ref.	ref.
Q2	0.992 (0.807, 1.220) 0.940	0.841 (0.660, 1.072) 0.165	0.801 (0.616, 1.042) 0.101
Q3	0.839 (0.676, 1.042) 0.115	0.688 (0.545, 0.870) 0.002	0.696 (0.541, 0.895) < 0.0001
Q4	0.442 (0.348, 0.561) < 0.0001	0.342 (0.258, 0.454) < 0.0001	0.352 (0.260, 0477) < 0.0001
*p* for trend	<0.0001	<0.0001	<0.0001
OBS Dietary	0.970 (0.958, 0.982) < 0.0001	0.956 (0.942, 0.970) < 0.0001	0.957 (0.942, 0.973) <0.0001
*OBS Dietary Quartile*			
Q1	ref.	ref.	ref.
Q2	1.020 (0.840, 1.241) 0.836	0.890 (0.703, 1.127) 0.336	0.802 (0.614,1.050) 0.111
Q3	0.908 (0.744,1.107) 0.339	0.751 (0.604, 0.934) 0.011	0.763 (0.593, 0.981) 0.037
Q4	0.568 (0.449, 0.718) < 0.0001	0.453 (0.341, 0.602) < 0.0001	0.446 (0.330, 0.604) < 0.001
*p* for trend	<0.0001	<0.0001	<0.0001
OBS Lifestyle	0.691 (0.656, 0.727) < 0.0001	0.676 (0.644, 0.709) < 0.0001	0.691 (0.656, 0.728) < 0.0001
*OBS Lifestyle Quartile*			
Q1	ref.	ref.	ref.
Q2	0.766 (0.597, 0.98) 0.037	0.762 (0.584, 0.994) 0.0466	0.780 (0.596, 1.020) 0.071
Q3	0.458 (0.365, 0.574) < 0.0001	0.430 (0.343, 0.540) < 0.0001	0.460 (0.366, 0.579) < 0.0001
Q4	0.226 (0.175, 0.291) < 0.0001	0.212 (0.163, 0.275) < 0.0001	0.226 (0.171, 0.299) < 0.0001
*p* for trend	<0.0001	<0.0001	<0.0001

OBS, oxidative balance score; NAFLD, nonalcoholic fatty liver disease; OR, odds ratio; 95% CI, 95% confidence interval; CVD, cardiovascular disease. Model 1 was an unadjusted model, model 2 adjusted for age, sex, race, marital status, education level, and daily energy intake, and model 3 was adjusted for age, sex, race, marital status, education level, daily energy intake, diabetes, hypertension, and CVD.

### Nonlinear relationship exploration

**Figure 3:**
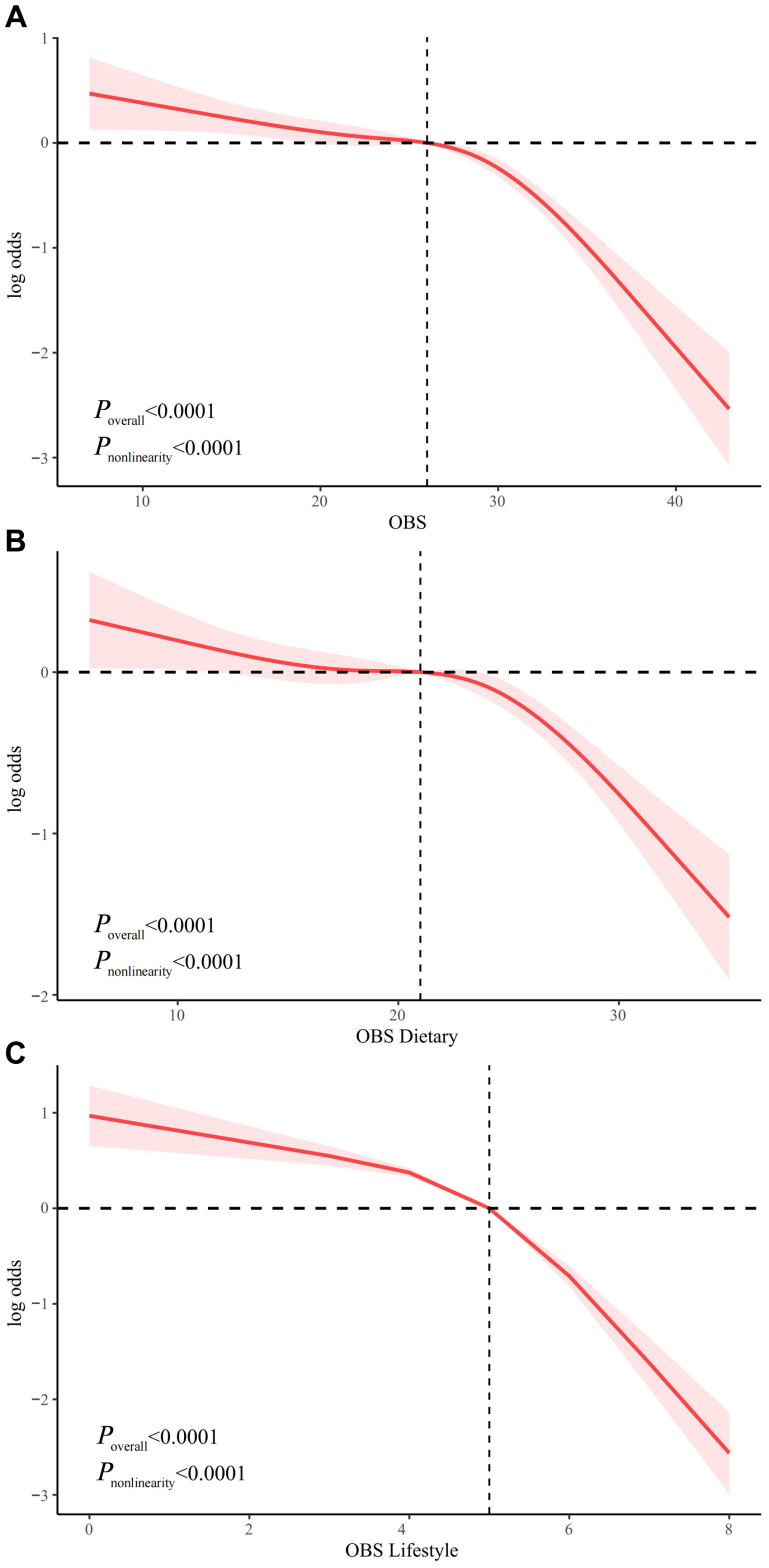
Non-linear relationship of all OBS with NAFLD. **(A)** Non-linear relationship between OBS and NAFLD. **(B)** Non-linear relationship between dietary OBS and NAFLD. **(C)** Non-linear relationship between lifestyle OBS and NAFLD. OBS, oxidative balance score; NAFLD, nonalcoholic fatty liver disease.

We examined the nonlinear relationship between OBS and risk of NAFLD using RCS models. OBS, dietary OBS, and lifestyle OBS were all nonlinearly associated with risk of NAFLD (all P nonlinearity <0.0001). In addition, the inflection points of the nonlinear curves for the relationship between OBS, dietary OBS, and lifestyle OBS and NAFLD were 26, 21, and 5, respectively (Figure 3). We therefore performed a threshold effect analysis based on the inflection point. Interestingly, OBS (both dietary and lifestyle OBS) was significantly negatively associated with NAFLD risk both before and after the inflection point. After the inflection point, OBS was more significantly associated with a reduced risk of NAFLD, suggesting that OBS, dietary OBS, and lifestyle OBS were associated with a more significant reduction in NAFLD risk after exceeding 26, 21, and 5 points, respectively (Supplementary Table S5).

### Mediation analysis

We then explored whether OBS indirectly affects NAFLD risk through markers related to inflammation, oxidative stress, and glycolipid metabolism. There was a significant direct effect of OBS with NAFLD risk in the presence of each individual mediating variable (all *p* < 0.0001). Indirect mediation effects were present for all included mediators (all *p* < 0.0001) except ALT and AST (all *p* > 0.05). The average mediated proportions of blood neutrophils, serum albumin, GGT, UA, bilirubin, TC, TG, HDL-cholesterol, and fasting glucose were 9.3, 7.6, 13.3, 13.9, 5.03, 0.9, 13.8, 29.3, and 3.6%, respectively. (All *p* < 0.0001, except for TC where *P* was 0.0120) (Table 4).

**Table 4:** Mediation analysis of the relationship between OBS and NAFLD.

	Direct effect (average)	Value of *p*	Mediation effect (average)	Value of *p*	Proportion-mediated (average)	*p*-value
Separate mediators						
Neutrophils	−0.116 (−0.133, −0.099)	<0.0001	−0.012 (−0.015, −0.009)	<0.0001	0.093 (0.066, 0.126)	<0.0001
ALT	−0.1120 (−0.141, −0.094)	<0.0001	−0.001 (−0.005, 0.003)	0.5740	0.009 (−0.024, 0.049)	0.5740
AST	−0.129 (−0.150, −0.106)	0.0060	0.002 (−0.0001, 0.008)	0.0540	−0.017 (−0.064, 0.004)	NA
Albumin	−0.117 (−0.134, −0.100)	<0.0001	−0.010 (−0.012, −0.007)	<0.0001	0.076 (0.054, 0.102)	<0.0001
GGT	−0.109 (−0.125, −0.090)	<0.0001	−0.017 (−0.025, −0.010)	<0.0001	0.133 (0.080, 0.197)	<0.0001
UA	−0.111 (−0.127, −0.094)	<0.0001	−0.018 (−0.022, −0.014)	<0.0001	0.139 (0.109, 0.177)	<0.0001
Bilirubin	−0.121 (−0.139, −0.103)	<0.0001	−0.006 (−0.009, −0.004)	<0.0001	0.0503 (0.032, 0.073)	<0.0001
TG	−0.111 (−0.129, −0.091)	<0.0001	−0.018 (−0.024, −0.009)	<0.0001	0.138 (0.071, 0.184)	<0.0001
TC	−0.127 (−0.143, −0.109)	<0.0001	−0.001 (−0.002, −0.0002)	0.0120	0.009 (0.002, 0.017)	0.0120
HDL-cholesterol	−0.091 (−0.107, −0.075)	<0.0001	−0.038 (−0.044, −0.032)	<0.0001	0.293 (0.246, 0.351)	<0.0001
Fasting glucose	−0.120 (−0.137, −0.101)	<0.0001	−0.005 (−0.008, −0.001)	<0.0001	0.036 (0.015, 0.069)	<0.0001

OBS, oxidative balance score; NAFLD, nonalcoholic fatty liver disease; NA, not available; ALT, alanine aminotransferase; AST, aspartate aminotransferase; GGT, gamma glutamyltransferase; UA, uric acid; TG, triglycerides; TC, total cholesterol; HDL, high-density lipoprotein.

### Stratified analysis

**Figure 4:**
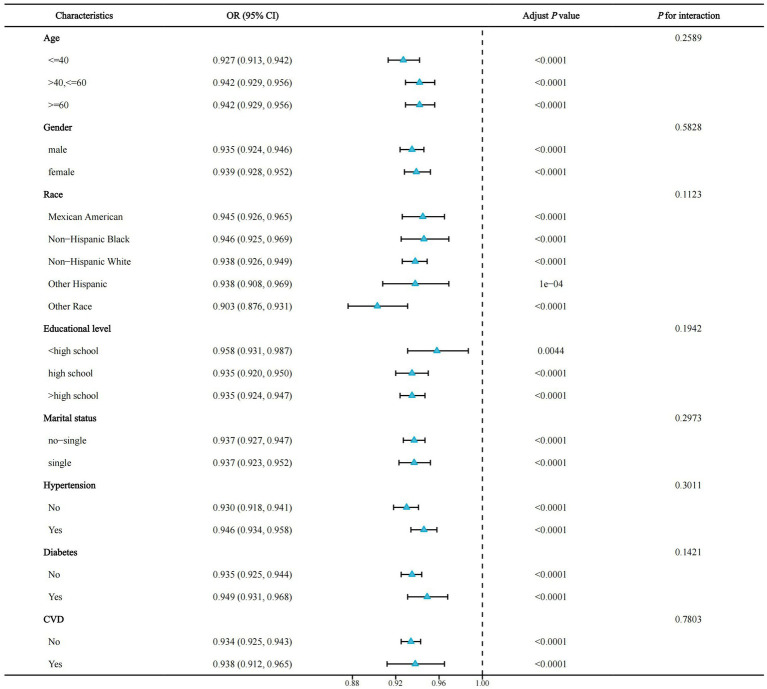
Forest plot for stratified analysis. OR, odds ratio; 95% CI, 95% confidence interval; CVD, cardiovascular disease.

To investigate whether the association of OBS with the risk of NAFLD was consistent across subgroups, we performed a stratified analysis. Notably, the *P* for interaction was >0.05 for all subgroups (age, sex, race, education level, marital status, diabetes, hypertension, and CVD), indicating that our findings were consistent across all subgroups (Figure 4).

### Sensitivity analysis

To verify the stability of our results, we performed a sensitivity analysis. We obtained similar results after dividing the OBS (including dietary and lifestyle OBS) by tertile or quintile (Supplementary Table S6). Second, we did not adjust for daily energy intake in the multivariate regression analysis. Consistently, the associations of OBS, dietary OBS, and lifestyle OBS with NAFLD all remained similarly significant (Supplementary Table S7). These results indicate a robust stability of our findings.

## Discussion

To the best of our knowledge, this study is the first to elucidate the effects of dietary and lifestyle modifiable pro- and antioxidants on NAFLD/AHF in a real-world setting using a comprehensive composite indicator of oxidative stress, the OBS. By analyzing data from a US nationally representative cross-sectional study over 20 years, we showed that higher OBS scores are nonlinearly associated with lower risk of NAFLD and that similar effects are present for independent dietary OBS and lifestyle OBS, supporting the critical impact of OBS on the onset and progression of NAFLD. Furthermore, through mediation analysis, we conclude that OBS may indirectly mediate NAFLD risk through effects on inflammation, oxidative stress, and glycolipid metabolism. Notably, OBS (both dietary OBS and lifestyle OBS) were not associated with the risk of AHF in patients with NAFLD.

Impaired oxidative stress signaling is closely related to the pathogenesis of NAFLD/NASH based on the critical mechanism of overproduction of ROS leading to cellular damage, senescence, and death, and can lead to disruption of lipid metabolism, impaired insulin signaling, and dysregulation of innate immune signaling pathways (26). Mechanistically, oxidative stress is essentially due to an imbalance between pro-oxidants (e.g., superoxide anions, hydroxyl radical, and hydrogen peroxide) and antioxidants (superoxide dismutase, glutathione peroxidase, and vitamins A, C, E, etc.) in the body (27). In the pathophysiology of NAFLD/NASH, lipotoxicity in the liver leads to impaired function of several ROS-generating organelles, such as mitochondria, endoplasmic reticulum, and peroxisomes, resulting in the release of abundant ROS and causing an imbalance in redox signaling. Accumulating experimental evidence suggests that modulation of redox signaling in NAFLD/NASH may attenuate disease progression and can serve as a potential target. Targeting nuclear factor E2-related factor 2, a redox-regulated transcription factor, has been demonstrated to improve NASH by ameliorating lipotoxicity, inflammation, and oxidative stress (28). In addition, scattered clinical data suggest that supplementation with a variety of dietary antioxidants such as vitamin E, vitamin C, and hesperidin, as well as lifestyle changes such as exercise, may improve clinical indicators in part by reducing oxidative stress in patients with NAFLD/NASH (29–31).

As there are still no approved drugs for the treatment of NAFLD/NASH, dietary and lifestyle changes remain the cornerstone of NAFLD management (32). Because of the current lack of real-world evidence on whether dietary and lifestyle pro- and antioxidants have integrated and independent effects on the onset and progression of NAFLD, we used a previously well-established composite score that assesses the pro- and antioxidants properties of components of dietary and lifestyle sources. The OBS is a tool used to assess overall redox homeostasis in individuals and has been shown to be associated with colorectal cancer, CVD, and chronic kidney disease in previous epidemiological studies (33). More recent studies using NHANES have suggested that higher OBS is associated with improved cognitive function and depressive symptoms, and reduced risk of periodontitis and diabetes compared with those with the lowest OBS (7, 8, 34, 35). After adjustment for all included confounders, OBS treated as both a continuous and a categorical variable, was associated with the risk of NAFLD but not AHF. Compared to Q1, the risk of NAFLD was reduced by approximately 65% in the population with OBS located in Q4 (*p* trend <0.0001). Notably, both higher dietary OBS and lifestyle OBS were also independently associated with reduced risk of NAFLD, with approximately 55 and 77% reductions in the Q4 population compared to the reference population, respectively. Thus, lifestyle OBS appears to be associated with a greater reduction in NAFLD risk than dietary OBS. The underlying mechanisms remain unclear, but a recent study using NHANES III showed that physical activity is more important than diet for the prognosis of patients with metabolic-associated fatty liver disease (MAFLD) and sarcopenia (36). Future mechanistic studies are needed to further explore the relevant mechanisms.

We then further investigated the nonlinear relationship between OBS and risk of NAFLD. Both independent and combined dietary OBS and lifestyle OBS were nonlinearly associated with the risk of NAFLD (*p* nonlinearity <0.0001). The inflection points for OBS, dietary OBS, and lifestyle OBS were 26, 21, and 5 points, respectively. We used threshold effect analysis to show that OBS, dietary OBS, and lifestyle OBS were associated with a more pronounced reductions in NAFLD risk after 26, 21 and 5 points, respectively, which may have important implications for the dietary and lifestyle management of patients with NAFLD.

We then hypothesized that OBS may exert beneficial effects indirectly through relevant markers. Therefore, we next investigated whether OBS indirectly reduces the risk of NAFLD via these pathways of inflammation, oxidative stress, and glycolipid metabolism. Not surprisingly, most of these potential biomarkers may mediate the protective effect of OBS against NAFLD, although ALT and AST do not appear to mediate the effect. HDL-cholesterol, UA, and TG may have mediated the highest proportions, at 29.3, 13.9, and 13.8%, respectively. HDL-cholesterol mediates the regulation of lipid metabolism mainly by promoting reverse cholesterol transport, antioxidant, and anti-inflammatory mechanisms, and previous studies have demonstrated that both diet and lifestyle management can improve HDL-cholesterol levels (37). Conversely, TG has been shown to be a core biomarker of lipid dysregulation in patients with NAFLD (24). Moreover, the role of UA in anti-oxidative stress has been well established. Our study suggests that OBS may indirectly reduce the risk of NAFLD mainly through these markers, and future studies are needed to further validate our findings.

We performed a stratified analysis to elicit whether OBS still had relevant effects in different subgroups. In all subgroups, we did not observe significant differences between groups (all *P* for interaction >0.05), suggesting the consistency of our study across subgroups and that OBS may reduce NAFLD risk in people with different characteristics.

To test the stability of our results, we performed a sensitivity analysis. After dividing OBS (including dietary OBS and lifestyle OBS) into tertiles and quintiles, we obtained similar significant negative correlations with NAFLD risk. Consistently, a similar effect remained after not adjusting for daily energy intake in multivariate regression analysis. These findings justify the stability of our results, and the conclusion that OBS is associated with a reduced risk of NAFLD is robust.

Our results show that in the real world, healthy dietary and lifestyle habits with higher antioxidant properties are associated with a significant reduction in the risk of NAFLD. In fact, healthy eating habits have been widely shown to be associated with a reduced risk of NAFLD/MAFLD and liver fibrosis. A variety of dietary quality indices, including the Dietary Inflammatory Index (DII), the Mediterranean diet, the Dietary Approach to Stop Hypertension, the Alternate Healthy Eating Index diet, and the Healthy Eating Index-2015 are associated with the risk of NAFLD/MAFLD and liver fibrosis, with the higher DII being associated with lower diet quality, while the rest of the indices are considered healthy eating indices (38–40). Lifestyle changes are also recognized as having a fundamental role in the improvement of NAFLD. Obesity is a major contributor to the development and progression of NAFLD and there is strong evidence that a 10% weight loss is effective in improving the histological characteristics of NASH patients (41). Physically active individuals have an approximately 30% reduced risk of NAFLD compared to inactive individuals (40). In addition, smoking and low-moderate alcohol consumption have been suggested to be associated with the development and severity of NAFLD (42–44). Our results highlight that both independent and combined healthy diet and lifestyle in terms of oxidative stress can significantly reduce the incidence and development of NAFLD.

Interestingly, OBS did not appear to be associated with progression of fibrosis in patients with NAFLD. An important randomized controlled clinical trial showed that vitamin E was associated with improved histological features including hepatic steatosis and lobular inflammation and reduced biochemical markers such as ALT and AST, but not with improvement in fibrosis, in patients with NASH (45). However, a recent meta-analysis concluded that vitamin E was associated with at least ≥1 fibrosis stage improvement compared with placebo (46). Using NHANES, a study showed an association between serum vitamin C, choline, alpha-carotene, and vitamin B12 and liver fibrosis (47). Similarly, other antioxidants may be associated with fibrosis improvement in patients with NAFLD (48). Currently, the underlying mechanisms remain unclear, but an important reason may be that OBS is a composite index that includes both pro-oxidants and antioxidants, and therefore its combined effect cannot be explained by the alleviation of fibrosis by antioxidants alone. Therefore, future high-quality prospective studies are needed to further confirm our findings.

A point that warrants the need for cautious interpretation is the possibility of selection bias and compromised generalizability of the results as our study population went from an initial population of 101,316 to a final population of 6,341. However, several explanations can be offered as follows. First, the exclusion criteria implemented in our study were all rigorous. The fasting blood glucose and insulin tests required for the diagnosis of USFLI were only performed in a subset of participants, yet this also ensured the reliability of the NAFLD diagnosis (compared to FLI) because the fasting blood tests excluded dietary interferences and ensured that the samples were fresh. We excluded the population with missing covariates, which is more likely to ensure the reliability of the results in subsequent regression analyses adjusting for confounders. Finally, and most importantly, our analyses were all appropriately weighted and considered complex sampling design according to the NHANES study guidelines (49), i.e., probabilistic sampling to ensure that the overall sample was represented by a smaller sample. Therefore, we believe that weighting all analyses to represent the overall population and rigorous exclusion criteria ensure the reliability and generalizability of the results. More large-sample studies are needed in the future to validate our conclusions.

Our study has several important strengths. First, we used a nationally representative population-based study to conduct our investigation, and the results are generalizable and representative. Second, we included possible covariates based on previous studies, which greatly reduced the effect of confounding factors. We further explored the direct and indirect effects of OBS on NAFLD using mediation analysis, which showed potential beneficial effects of OBS through inflammation, oxidative stress, and glycolipid metabolism. Stratified and sensitivity analyses highlighted the robustness and stability of our findings. However, our study also has limitations. First, our study is a cross-sectional study, and thus residual confounders may still exist, and causal relationships cannot be drawn. Second, our study was conducted in the US population, and findings for other races and countries need to be further explored in future studies. Finally, we diagnosed NAFLD/AHF using non-invasive markers rather than liver biopsy, and thus may lack accuracy. However, because of the cost of liver biopsy, the potential risks of the operation, and the inability to generalize in large population-based studies, as well as the proven good accuracy of non-invasive scores, diagnosis using non-invasive markers is therefore also well characterized and these effects are unlikely to affect the reliability of the results.

## Conclusion

Dietary and lifestyle OBS had joint and independent protective effects on the risk of NAFLD in a nationally representative cross-sectional study and showed a non-linear relationship. However, no association was found between OBS and AHF risk. The population with OBS in the highest quartile had a 65% reduction in NAFLD risk compared to the lowest quartile. OBS may indirectly affect the development of NAFLD through inflammation, oxidative stress, and glycolipid metabolism. These findings may be instrumental in stratifying the risk of NAFLD in the general population and allowing for interventions.

## Data availability statement

The raw data supporting the conclusions of this article will be made available by the authors, without undue reservation.

## Ethics statement

The studies involving humans were approved by The National Center for Health Statistics (NCHS) Research Ethics Review Board. The studies were conducted in accordance with the local legislation and institutional requirements. The participants provided their written informed consent to participate in this study.

## Author contributions

YL: Conceptualization, Formal analysis, Software, Writing – original draft. MC: Supervision, Writing – review & editing.

## Footnotes

^1^ https://wwwn.cdc.gov/nchs/nhanes/Default.aspx

## Glossary

NAFLD: Nonalcoholic fatty liver disease
AHF: Advanced liver fibrosis
OBS: Oxidative balance score
ROS: Reactive oxygen species
NHANES: National Health and Nutrition Examination Survey
NCHS: National Center for Health Statistics
USFLI: US fatty liver index
BMI: Body mass index
GGT: Gamma glutamyltransferase
WC: Waist circumference
NFS: NAFLD fibrosis score
FIB-4: Fibrosis 4 index
AST: Aspartate aminotransferase
APRI: AST/platelet ratio index
ALT: Alanine aminotransferase
ULN: Upper limit of normal
PIR: Family income to poverty ratio
CVD: Cardiovascular disease
UA: Uric acid
TG: Triglycerides
TC: Total cholesterol
HDL: High-density lipoprotein
95% CI: 95% confidence interval
RCS: Restricted cubic splines
OR: Odds ratio
DII: Dietary Inflammatory Index
