# Supplementary material for: Dietary and lifestyle oxidative balance scores are independently and jointly associated with nonalcoholic fatty liver disease: a 20 years nationally representative cross-sectional study
Source: Front Nutr. 2023 Oct 18;10:1276940. doi: 10.3389/fnut.2023.1276940 (PMC10619002; doi:10.3389/fnut.2023.1276940)
Supplement: Supplementary file 1 [file Data_Sheet_1.docx]

**Supplementary table 1.** Description of the components of OBS.

| **OBS components** | **Antioxidant or pro-oxidant** |
| --- | --- |
| **Dietary OBS** | |
| Dietary fiber | Antioxidant |
| Carotene | Antioxidant |
| Vitamin B2 | Antioxidant |
| Niacin | Antioxidant |
| Vitamin B6 | Antioxidant |
| Total folate | Antioxidant |
| Vitamin B12 | Antioxidant |
| Vitamin C | Antioxidant |
| Vitamin E | Antioxidant |
| Calcium | Antioxidant |
| Magnesium | Antioxidant |
| Zinc | Antioxidant |
| Copper | Antioxidant |
| Selenium | Antioxidant |
| Total fat | Pro-oxidant |
| Iron | Pro-oxidant |
| **Lifestyle OBS** |  |
| Physical activity | Antioxidant |
| Alcohol | Pro-oxidant |
| Body mass index | Pro-oxidant |
| Cotinine | Pro-oxidant |

Abbreviations: OBS, oxidative balance score.

**Supplementary table 2**. Baseline characteristics classified according to NAFLD status.

|  | **Total** | **NO-NAFLD** | **NAFLD** | **P-value** |
| --- | --- | --- | --- | --- |
| **Sample N** | n=6341 | n=4317 | n=2024 |  |
| **Weighted N** | N=129,919,267 | N=90,675,162 | N=39,244,105 |  |
| **Age** | 47.516 (46.560,48.472) | 45.539 (44.369,46.709) | 52.085 (51.358,52.811) | <0.0001 |
| **PIR** | 3.302 (3.203,3.401) | 3.317 (3.187,3.448) | 3.265 (3.156,3.374) | 0.5306 |
| **OBS Dietary** | 20.784 (20.426,21.142) | 21.223 (20.786,21.660) | 19.769 (19.305,20.233) | <0.0001 |
| **OBS** | 25.266 (24.871,25.661) | 25.968 (25.497,26.439) | 23.644 (23.133,24.156) | <0.0001 |
| **OBS Lifestyle** | 4.482 (4.410,4.555) | 4.745 (4.671,4.820) | 3.875 (3.754,3.997) | <0.0001 |
| **Daily energy intake**(**kcal/day)** | 1992.522 (1952.935 ,2032.110) | 1976.082 (1932.452 ,2019.713) | 2030.507 (1967.533 ,2093.481) | 0.1157 |
| **Gender** |  |  |  | <0.0001 |
| Male | 52.517 (50.907,54.121) | 48.182 (45.979,50.393) | 62.531 (59.483,65.484) |  |
| Female | 47.483 (45.879,49.093) | 51.818 (49.607,54.021) | 37.469 (34.516,40.517) |  |
| **Race** |  |  |  | <0.0001 |
| Mexican American | 5.389 (4.604,6.298) | 4.101 (3.484,4.823) | 8.364 (6.899,10.107) |  |
| Non-Hispanic Black | 9.098 (7.717,10.697) | 10.662 (9.079,12.484) | 5.482 (4.323,6.930) |  |
| Non-Hispanic White | 74.868 (72.628,76.982) | 74.070 (71.706,76.302) | 76.710 (73.584,79.569) |  |
| Other Hispanic | 4.245 (3.121,5.750) | 4.320 (3.085,6.018) | 4.072 (2.858,5.771) |  |
| Other race | 6.401 (5.398,7.574) | 6.846 (5.704,8.197) | 5.372 (3.956,7.256) |  |
| **Marital** |  |  |  | 0.0136 |
| Single | 73.223 (71.049,75.290) | 71.461 (68.419,74.319) | 77.293 (74.056,80.235) |  |
| No single | 26.777 (24.710,28.951) | 28.539 (25.681,31.581) | 22.707 (19.765,25.944) |  |
| **Diabetes** |  |  |  | <0.0001 |
| No | 88.883 (87.541,90.097) | 95.184 (94.233,95.985) | 74.324 (71.490,76.966) |  |
| Yes | 11.117 (9.903,12.459) | 4.816 (4.015,5.767) | 25.676 (23.034,28.510) |  |
| **Hypertension** |  |  |  | <0.0001 |
| No | 64.871 (63.073,66.628) | 73.586 (71.139,75.895) | 44.736 (40.871,48.667) |  |
| Yes | 35.129 (33.372,36.927) | 26.414 (24.105,28.861) | 55.264 (51.333,59.129) |  |
| **CVD** |  |  |  | <0.0001 |
| No | 92.580 (91.465,93.559) | 94.877 (93.932,95.681) | 87.272 (84.967,89.268) |  |
| Yes | 7.420 (6.441,8.535) | 5.123 (4.319,6.068) | 12.728 (10.732,15.033) |  |
| **Education level** |  |  |  | 0.0007 |
| Under high school | 4.273 (3.571,5.104) | 3.410 (2.636,4.401) | 6.266 (5.028,7.783) |  |
| High school | 31.702 (28.924,34.618) | 30.339 (27.116,33.768) | 34.852 (30.564,39.401) |  |
| More than high school | 64.025 (61.187,66.769) | 66.251 (62.805,69.533) | 58.882 (54.476,63.149) |  |

Abbreviations: OBS, oxidative balance score; NAFLD, nonalcoholic fatty liver disease; PIR, family income to poverty ratio; CVD, cardiovascular disease. For continuous variables: survey-weighted mean (95% CI), P-value was by survey-weighted linear regression. For categorical variables: survey-weighted percentage (95% CI), P-value was by survey-weighted Chi-square test.

**Supplementary table 3**. Baseline characteristics classified according to AHF status.

|  | **Total** | **NO-AHF** | **AHF** | **P-value** |
| --- | --- | --- | --- | --- |
| **Sample N** | n=1917 | n=1651 | n=266 |  |
| **Weighted N** | N=39244106 | N=35313049 | N=3931057 |  |
| **Age** | 52.085(51.358,52.811) | 51.020(50.287,51.753) | 61.650(59.105,64.196) | <0.0001 |
| **PIR** | 3.265(3.156,3.374) | 3.291(3.181,3.400) | 3.034(2.766,3.303) | 0.056 |
| **OBS** | 23.644(23.133,24.156) | 23.785(23.254,24.317) | 22.377(21.181,23.574) | 0.029 |
| **OBS dietary** | 19.769(19.305,20.233) | 19.907(19.417,20.396) | 18.533(17.416,19.649) | 0.025 |
| **OBS lifestyle** | 3.875(3.754,3.997) | 3.879(3.754,4.004) | 3.845(3.597,4.093) | 0.786 |
| **Daily energy intake**(**kcal/day)** | 2030.507(32.130) | 2066.103(34.370) | 1710.749(57.818) | <0.0001 |
| **Gender** |  |  |  | 0.075 |
| Male | 62.531(56.170,68.893) | 63.291(60.142,66.439) | 55.709(47.578,63.841) |  |
| Female | 37.469(32.300,42.637) | 36.709(33.561,39.858) | 44.291(36.159,52.422) |  |
| **Race** |  |  |  | 0.01 |
| Mexican American | 8.364(6.833, 9.894) | 8.623(6.919,10.327) | 6.037(3.505, 8.569) |  |
| Non-Hispanic Black | 5.482(4.394, 6.571) | 5.107(3.828, 6.386) | 8.860(4.931,12.788) |  |
| Non-Hispanic White | 76.710(67.359,86.061) | 76.387(73.251,79.522) | 79.616(74.737,84.494) |  |
| Other Hispanic | 4.072(2.689, 5.454) | 4.272(2.724,5.820) | 2.272(1.005,3.540) |  |
| Other race | 5.372(3.616, 7.128) | 5.612(3.829,7.395) | 3.215(1.122,5.308) |  |
| **Marital** |  |  |  | 0.012 |
| Single | 77.293(68.461,86.126) | 78.351(75.031,81.670) | 67.797(59.729,75.865) |  |
| No single | 22.707(19.312,26.101) | 21.649(18.330,24.969) | 32.203(24.135,40.271) |  |
| **Education level** |  |  |  | 0.029 |
| Under high school | 6.266(4.867, 7.665) | 6.119(4.617, 7.620) | 7.589(3.563,11.614) |  |
| High school | 34.852(28.786,40.918) | 33.802(29.086,38.517) | 44.292(36.152,52.432) |  |
| More than high school | 58.882(52.118,65.646) | 60.080(55.690,64.470) | 48.119(39.601,56.637) |  |
| **Diabetes** |  |  |  | <0.0001 |
| No | 74.324(65.620,83.028) | 78.074(75.373,80.776) | 40.631(29.363,51.899) |  |
| Yes | 25.676(22.683,28.669) | 21.926(19.224,24.627) | 59.369(48.101,70.637) |  |
| **Hypertension** |  |  |  | 0.005 |
| No | 44.736(39.323,50.150) | 46.268(42.276,50.260) | 30.978(21.033,40.924) |  |
| Yes | 55.264(48.082,62.445) | 53.732(49.740,57.724) | 69.022(59.076,78.967) |  |
| **CVD** |  |  |  | <0.0001 |
| No | 87.272(77.969,96.575) | 89.255(86.896,91.614) | 69.458(62.150,76.767) |  |
| Yes | 12.728(10.479,14.977) | 10.745(8.386,13.104) | 30.542(23.233,37.850) |  |

Abbreviations: OBS, oxidative balance score; AHF, advanced liver fibrosis; PIR, family income to poverty ratio; CVD, cardiovascular disease. For continuous variables: survey-weighted mean (95% CI), P-value was by survey-weighted linear regression. For categorical variables: survey-weighted percentage (95% CI), P-value was by survey-weighted Chi-square test.

**Supplementary table 4**. Multivariate regression analysis of OBS and AHF risk.

|  | **Model 1**  **OR (95% CI), P** | **Model 2**  **OR (95% CI), P** | **Model 3**  **OR (95% CI), P** |
| --- | --- | --- | --- |
| **OBS** | 0.970 (0.952, 0.988) 0.0029 | 0.989 (0.967, 1.011) 0.3260 | 0.987 (0.964, 1.010) 0.2692 |
| **OBS quartile** |  |  |  |
| Q1 | ref. | ref. | ref. |
| Q2 | 0.945 (0.661, 1.350) 0.7541 | 0.978 (0.666, 1.437) 0.9114 | 0.905 (0.606, 1.350) 0.6236 |
| Q3 | 0.833 (0.575, 1.205) 0.3313 | 1.065 (0.710, 1.597) 0.7618 | 1.009 (0.662, 1.539) 0.9663 |
| Q4 | 0.556 (0.378, 0.818) 0.0029 | 0.803 (0.516, 1.248) 0.3294 | 0.762 (0.481, 1.208) 0.2478 |
| **p for trend** | 0.0019 | 0.4381 | 0.3628 |

Abbreviations: OBS, oxidative balance score; AHF, advanced liver fibrosis; OR, odds ratio; 95% CI, 95% confidence interval. Model 1 was an unadjusted model, model 2 adjusted for age, sex, race, marital status, education level, and daily energy intake, and model 3 was fully adjusted.

**Supplementary table 5**. Threshold effect analysis of the nonlinear relationship between OBS and NAFLD.

|  | **Model 1**  **OR (95% CI), P** | **Model 2**  **OR (95% CI), P** | **Model 3**  **OR (95% CI), P** |
| --- | --- | --- | --- |
| **OBS≤26** | 0.981 (0.967, 0.996) 0.016 | 0.962(0.946, 0.979) <0.0001 | 0.962 (0.945,0.980) <0.0001 |
| **OBS> 26** | 0.903(0.882, 0.924) <0.0001 | 0.885(0.863, 0.909) <0.0001 | 0.889 (0.865, 0.914) <0.0001 |
| **OBS dietary ≤21** | 0.985 (0.968, 1.002) 0.0898 | 0.968 (0.949, 0.987) 0.0013 | 0.967 (0.947, 0.988) 0.0018 |
| **OBS dietary >21** | 0.915 (0.893, 0.938) <0.0001 | 0.899 (0.875, 0.923) <0.0001 | 0.756 (0.605, 0.945) 0.0141 |
| **OBS lifestyle ≤5** | 0.692(0.668, 0.718) <0.0001 | 0.652(0.626, 0.679) <0.0001 | 0.663(0.635, 0.692) <0.0001 |
| **OBS lifestyle >5** | 0.385 (0.302, 0.490) <0.0001 | 0.358 (0.277, 0.461) <0.0001 | 0.366 (0.279, 0.479) <0.0001 |

Abbreviations: OBS, oxidative balance score; NAFLD, nonalcoholic fatty liver disease; OR, odds ratio; 95% CI, 95% confidence interval. Model 1 was an unadjusted model, model 2 adjusted for age, sex, race, marital status, education level, and daily energy intake, and model 3 was fully adjusted.

**Supplementary table 6**. Multivariate regression analysis with OBS division according to tertiles or quintiles.

|  | **Model 1**  **OR (95% CI), P** | **Model 2**  **OR (95% CI), P** | **Model 3**  **OR (95% CI), P** |
| --- | --- | --- | --- |
| **OBS** |  |  |  |
| T1 | ref. | ref. | ref. |
| T2 | 0.801 (0.704, 0.912) 0.0008 | 0.840 (0.694, 1.015)0.073 | 0.834(0.683, 1.017) 0.075 |
| T3 | 0.482 (0.423, 0.549) <0.0001 | 0.429 (0.341, 0.539) <0.0001 | 0.440(0.341, 0.567) <0.0001 |
| ***p for trend*** | <0.0001 | <0.0001 | <0.0001 |
| Q1 | ref. | ref. | ref. |
| Q2 | 0.809 (0.688, 0.951) 0.0101 | 0.715 (0.602, 0.850) 0.0001 | 0.832(0.591, 1.172) 0.295 |
| Q3 | 0.763 (0.646, 0.899) 0.0013 | 0.621 (0.519, 0.743) <0.0001 | 0.763 (0.592, 0.982) 0.038 |
| Q4 | 0.597 (0.508, 0.701) <0.0001 | 0.456 (0.380, 0.548) <0.0001 | 0.552 (0.400, 0.761) 0.0004 |
| Q5 | 0.332 (0.278, 0.397) <0.0001 | 0.233 (0.189, 0.287) <0.0001 | 0.293 (0.209, 0.411) <0.0001 |
| ***p for trend*** | <0.0001 | <0.0001 | <0.0001 |
| **OBS dietary** |  |  |  |
| T1 | ref. | ref. | ref. |
| T2 | 0.872 (0.768, 0.989) 0.0332 | 0.789 (0.687, 0.905) 0.0008 | 0.806 (0.695, 0.935) 0.0043 |
| T3 | 0.552 (0.484, 0.631) <0.0001 | 0.463 (0.395, 0.543) <0.0001 | 0.462 (0.391, 0.548) <0.0001 |
| ***p for trend*** | <0.0001 | <0.0001 | <0.0001 |
| Q1 | ref. | ref. | ref. |
| Q2 | 1.022 (0.864, 1.209) 0.7978 | 0.917 (0.767, 1.096) 0.3402 | 0.887 (0.733, 1.075) 0.2217 |
| Q3 | 0.856 (0.722, 1.016) 0.0746 | 0.732 (0.608, 0.882) 0.0010 | 0.742 (0.608, 0.905) 0.0032 |
| Q4 | 0.771 (0.648, 0.917) 0.0033 | 0.630 (0.518, 0.765) <0.0001 | 0.647 (0.526, 0.796) <0.0001 |
| Q5 | 0.504 (0.425, 0.598) <0.0001 | 0.387 (0.316, 0.475) <0.0001 | 0.392 (0.316, 0.486) <0.0001 |
| ***p for trend*** | <0.0001 | <0.0001 | <0.0001 |
| **OBS lifestyle** |  |  |  |
| T1 | ref. | ref. | ref. |
| T2 | 0.788 (0.678, 0.915) 0.0018 | 0.732 (0.624, 0.857) 0.0001 | 0.746 (0.631, 0.881) 0.0006 |
| T3 | 0.349 (0.306, 0.399) <0.0001 | 0.293 (0.254, 0.338) <0.0001 | 0.310 (0.266, 0.360) <0.0001 |
| ***p for trend*** | <0.0001 | <0.0001 | <0.0001 |
| Q1 | ref. | ref. | ref. |
| Q2 | 0.767 (0.618, 0.952) 0.0163 | 0.742 (0.590, 0.933) 0.0106 | 0.733 (0.576, 0.932) 0.0114 |
| Q3 | 0.672 (0.551, 0.819) <0.0001 | 0.606 (0.491, 0.748) <0.0001 | 0.613 (0.491, 0.765) <0.0001 |
| Q4 | 0.445 (0.364, 0.544) <0.0001 | 0.371 (0.299, 0.460) <0.0001 | 0.382 (0.305, 0.478) <0.0001 |
| Q5 | 0.208 (0.170, 0.255) <0.0001 | 0.163 (0.131, 0.204) <0.0001 | 0.174 (0.138, 0.219) <0.0001 |
| ***p for trend*** | <0.0001 | <0.0001 | <0.0001 |

Abbreviations: OBS, oxidative balance score; OR, odds ratio; 95% CI, 95% confidence interval. Model 1 was an unadjusted model, model 2 adjusted for age, sex, race, marital status, education level, and daily energy intake, and model 3 was fully adjusted.

**Supplementary table 7**. Multivariate regression analysis of OBS and risk of NAFLD without adjustment for daily energy intake.

|  | **Model 1**  **OR (95% CI), P** | **Model 2**  **OR (95% CI), P** | **Model 3**  **OR (95% CI), P** |
| --- | --- | --- | --- |
| **OBS** | 0.960(0.950,0.971) <0.0001 | 0.957(0.946, 0.967) <0.0001 | 0.962(0.951, 0.974) <0.0001 |
| **OBS Quartile** |  |  |  |
| Q1 | ref. | ref. | ref. |
| Q2 | 0.992(0.807,1.220) 0.940 | 0.919(0.729,1.158) 0.474 | 0.900(0.702,1.153) 0.405 |
| Q3 | 0.839(0.676,1.042) 0.115 | 0.794(0.621,1.013) 0.065 | 0.837(0.647,1.082) 0.177 |
| Q4 | 0.442(0.348,0.561) <0.0001 | 0.426(0.335,0.540) <0.0001 | 0.466(0.364,0.596) <0.001 |
| ***p for trend*** | <0.0001 | <0.0001 | <0.0001 |
| **OBS Dietary** | 0.970(0.958,0.982) <0.0001 | 0.967(0.955, 0.980) <0.0001 | 0.973(0.960,0.986) <0.0001 |
| **OBS Dietary Quartile** |  |  |  |
| Q1 | ref. | ref. | ref. |
| Q2 | 1.020(0.840,1.241) 0.836 | 0.965(0.773, 1.204) 0.752 | 0.901(0.703,1.153) 0.407 |
| Q3 | 0.908(0.744,1.107) 0.339 | 0.856(0.688, 1.066) 0.168 | 0.914(0.711,1.175) 0.483 |
| Q4 | 0.568(0.449,0.718) <0.0001 | 0.552(0.438,0.694) <0.0001 | 0.585(0.460,0.743) <0.001 |
| ***p for trend*** | <0.0001 | <0.0001 | 0.0001 |
| **OBS Lifestyle** | 0.691(0.656,0.727) <0.0001 | 0.676(0.644,0.709) <0.0001 | 0.691(0.656,0.728) <0.0001 |
| **OBS Lifestyle Quartile** |  |  |  |
| Q1 | ref. | ref. | ref. |
| Q2 | 0.766(0.597,0.98) 0.037 | 0.762(0.584,0.994) 0.0466 | 0.780(0.596,1.020) 0.071 |
| Q3 | 0.458(0.365,0.574) <0.0001 | 0.430(0.343,0.540) <0.0001 | 0.460(0.366,0.579) <0.0001 |
| Q4 | 0.226(0.175,0.291) <0.0001 | 0.212(0.163,0.275) <0.0001 | 0.226(0.171,0.299) <0.0001 |
| ***p for trend*** | <0.0001 | <0.0001 | <0.0001 |

Abbreviations: OBS, oxidative balance score; NAFLD, nonalcoholic fatty liver disease; OR, odds ratio; 95% CI, 95% confidence interval. Model 1 was an unadjusted model, model 2 adjusted for age, sex, race, marital status, and education level, and model 3 was adjusted for age, sex, race, marital status, education level, diabetes, hypertension, and CVD.
